# Radiation-induced fibrosarcoma after breast-conserving therapy for breast cancer: a case report and literature review

**DOI:** 10.1186/s40792-023-01629-4

**Published:** 2023-03-29

**Authors:** Hideko Hoshina, Kouichi Kubouchi, Yutaka Tsutsumi, Hiroyuki Takei

**Affiliations:** 1 Department of Breast Surgery and Oncology, Nippon Medical School, 1-1-5 Sendagi, Bunkyo-Ku, Tokyo, 113-8602 Japan; 2 Department of Breast Surgery, Kikuna Memorial Hospital, 4-4-27 Kikuna, Kouhoku-Ku, Yokohama, Kanagawa 222-0011 Japan; 3 Diagnostic Pathology Clinic, Pathos Tsutsumi, 1551-1 Miyoshiato, Yawase-Cho, Inazawa, Aichi 492-8342 Japan

**Keywords:** Fibrosarcoma, Breast cancer, Radiation therapy, Radiation-induced sarcoma, Radiation-induced fibrosarcoma

## Abstract

**Background:**

Radiation-induced sarcoma (RIS) has a 10-year incidence of 0.2–0.27% and a poor prognosis, although the radiation should need for breast-conserving surgery. In particular, radiation-induced fibrosarcoma has been rarer, and its incidence is 2.6–3.7% of RIS.

**Case presentation:**

A 43-year-old woman with pT1N1M0 breast cancer underwent breast-conserving surgery, chemotherapy, radiation therapy 8 years ago, and continued hormonal therapy. She complained of a hard unprotruded mass palpated in her right upper outer quadrant of breast. Although we suspected local recurrence, core needle biopsy revealed atypical spindled tumor cells without mammary or epithelial markers. A diagnosis of fibrosarcoma was made via tumorectomy. She underwent additional enlarged surgery.

**Conclusions:**

We report a rare case of fibrosarcoma in breast after breast-conserving surgery and radiation therapy. Fibrosarcoma after radiation therapy for breast cancer has been reported in 30 cases, including the present case. The dead and alive cases were not significantly different in terms of age, primary breast cancer, radiation dose, and following months. Patients with breast masses after radiation therapy should be suspected local recurrence and RIS.

## Background

The effects of radiation after breast-conserving surgery absolutely reduce each 10-year recurrence risk or 15-year risk of breast cancer death [1]. However, radiation therapy has also been reported to cause adverse events. Anorexia, malaise, and dermatitis occur in the acute phase. In the sub-acute or late phase, there is pneumonia, cardiotoxicity, anetoderma, and secondary cancer [2, 3]. As secondary cancer, radiation-induced sarcoma (RIS) has a 10-year incidence of 0.2–0.27% [4] and poor prognosis with a 5-year actuarial survival of 36–41% [4, 5]. A collaborative group of early breast cancer trialists reported a ratio of rates of 2.34 (2*p* = 0.03) of soft-tissue sarcoma after radiation in their meta-analysis [6]. In particular, radiation-induced fibrosarcoma has been rarer, and its incidence is 2.6–3.7% of RIS [4, 7].

We report a rare case of fibrosarcoma after breast-conserving surgery and radiation therapy and review and discuss radiation-induced fibrosarcomas after breast cancer which had been reported.

## Case presentation

**Fig. 1:**
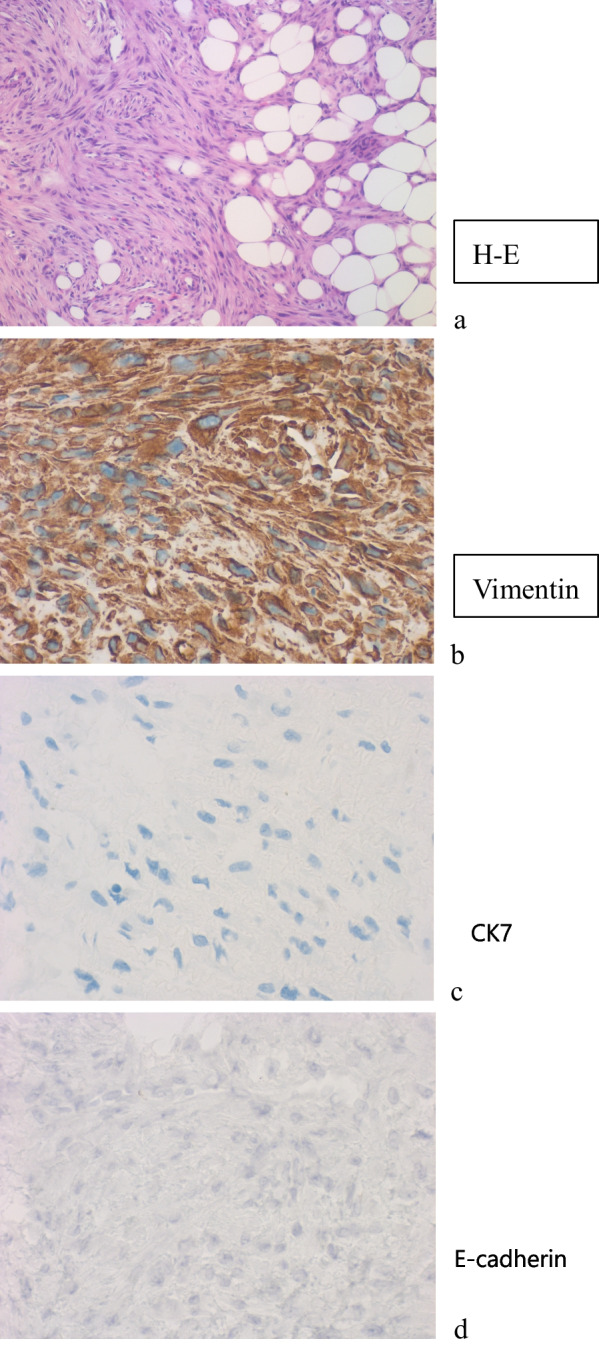
Pathology of core needle biopsy suspected fibrosarcoma. Core needle biopsy revealed atypical spindled tumor cells (**a**) with Vimentin expression highly positive (**b**). Epithelial markers CK7 (**c**) and E-cadherin (**d**) are negative

**Fig. 2:**
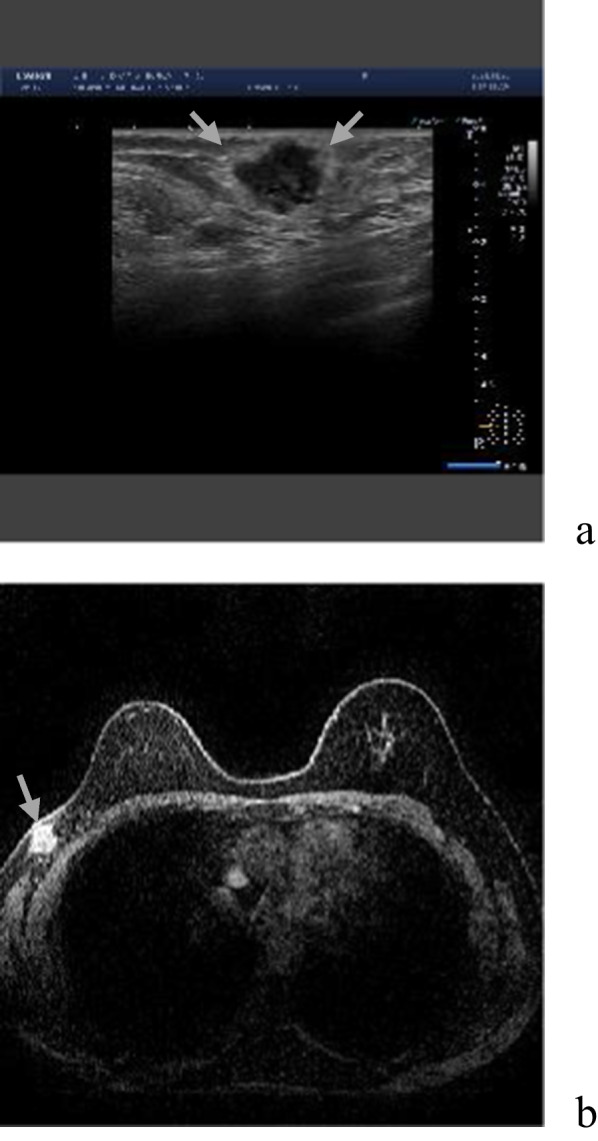
Ultrasonography and magnetic resonance imaging of the tumor. Ultrasonography scans revealed a 21.3-mm tumor in breast (**a**). Magnetic resonance imaging revealed 16 mm irregular geometries close to the skin (**b**)

A 43-year-old Japanese woman visited our outpatient clinic with a right axillary mass. She had a medical history of right breast cancer for 8 years. The primary histology was an 18-mm invasive ductal carcinoma in lower inner quadrant of the right breast with two lymph node metastases, which had hormone receptors and lacked human epidermal growth factor receptor 2 (HER2) amplification. The patient underwent breast-conserving surgery and axillary dissection, diagnosed pathological stage IIA (T1N1M0), and administrated chemotherapy with docetaxel and cyclophosphamide, and radiation therapy (50.0 Gy). During the administration of planned 10-year-tamoxifen and terminated 5-year-luteinizing hormone-releasing hormone agonist, a hard mass of 8.4 mm palpated in her right upper outer quadrant of breast without ulcered and protruded lesions, while no other abnormal findings were identified. Mammography revealed normal breast tissue. We suspected local recurrence of breast cancer. Findings of a core needle biopsy (CNB) revealed a proliferation of fibroblasts, but the lesion was judged to be benign. The lesion had grown for 7 months. After CNB was added, atypical spindled tumor cells without breast cancer markers (hormone receptor, HER2, and FOXA1) and epithelial markers (EMA, E-cadherin, and cytokeratin7, 8, 18, 20, AE1/AE3) were observed. As a mesenchymal marker, vimentin is highly expressed (Table 1, Fig. 1). Radiographic tests were negative for metastasis, ultrasonography scans revealed a 21.3 mm tumor with much vascular flow (Fig. 2a), and magnetic resonance imaging revealed 16-mm irregular geometries close to the skin (Fig. 2b). We decided to perform a tumorectomy to confirm the diagnosis.

**Table 1:** Immunostaining results for breast, mesenchymal, and epithelial markers

Breast markers	Epithelial markers		Mesenchymal markers	
Negative	Positive	Negative	Positive	Negative
ER	–	CK 7	Vimentin (strongly)	Desmin
PgR		CK 8	SMA (very weakly)	
AR		CK 18		
FOXA1		CK 20		
HER2		CK AE1/AE3		
P53		CK 34B-E12		
		EMA		
		E-cadherin		

*ER* estrogen receptor, *PgR* progesterone receptor, *AR* androgen receptor, *FOXA1* Forkhead box protein A1, *HER2* human epidermal growth factor receptor2, *CK* cytokeratin, *SMA* smooth muscle actin

**Fig. 3:**
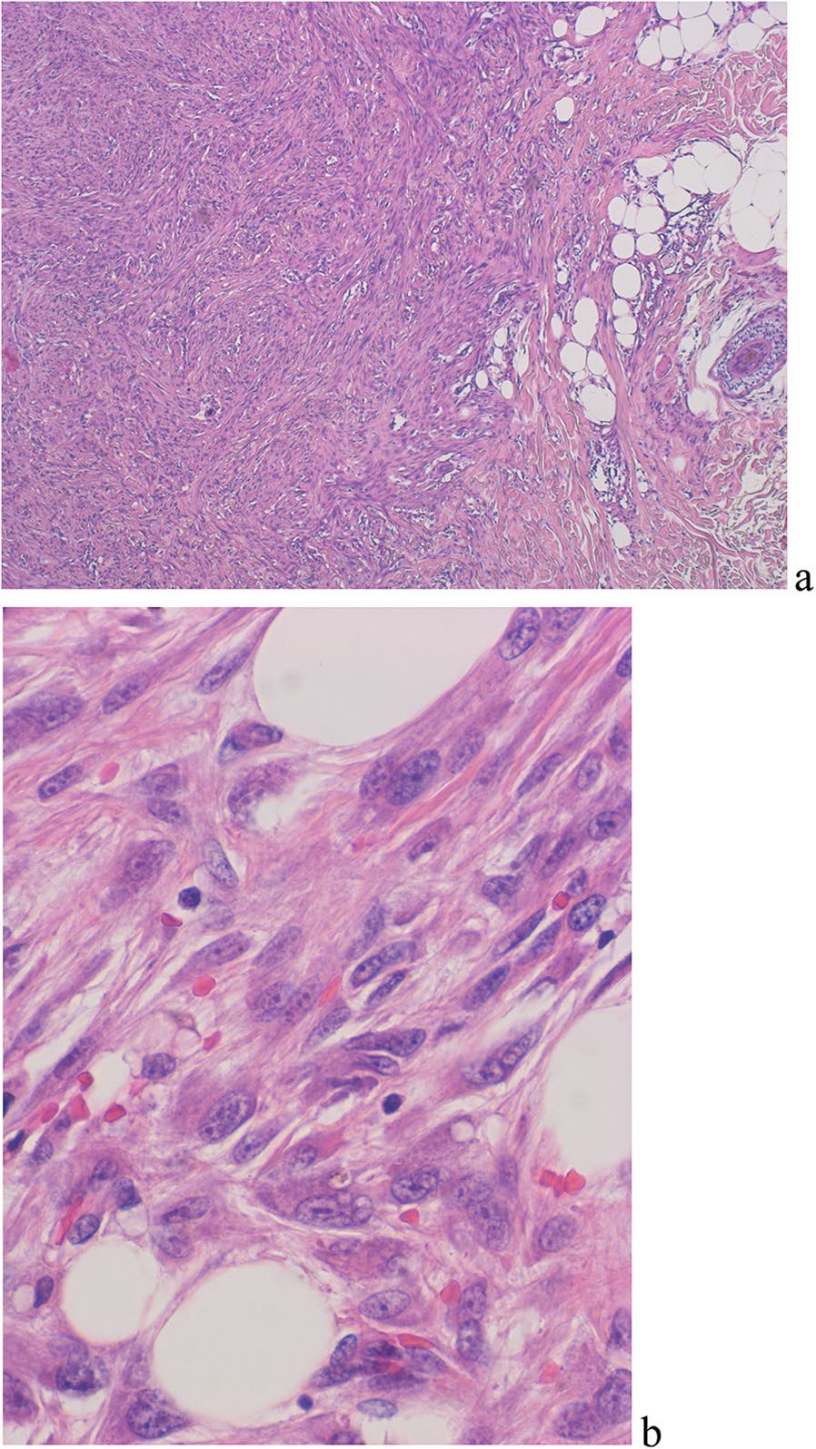
Pathology of surgical specimen revealed fibrosarcoma. Atypical spindled cells involved in subcutaneous of HE expression; low-power field (**a**), high-power field (**b**)

The pathological diagnosis revealed an 18 mm subcutaneous fibrosarcoma of the adult classic type close to breast (Fig. 3). In addition, skin excision was performed because the surgical margin of the skin was pathologically positive, leading to negative skin margins. The patient received a second opinion at the National Cancer Center Hospital and underwent enlarged excision there.

## Discussion and conclusion

The patients performed breast-conserving surgery and radiation had an isolated local recurrence risk reported on 13.1% for 10 years [6], which was higher than RIS incidence. In 1948, RIS was defined as having a history of radiation therapy, occurring in or near the radiation field, and being histologically different from primary cancer [8]. In breast-conserving therapy for breast cancer, angiosarcoma of RIS has the highest reported standardized incidence rations 26.2 [9]. Radiation therapy for breast cancer involves RIS of the chest wall, pleura, and upper extremity [10, 11]. RIS has a poorer prognosis in patients over 60 years, high-grade tumors, and positive margins [5]. RIS in cutaneous was likely to occur as protruded mass and to have relatively a good prognosis [9].

Radiation therapy damages deoxyribonucleic acid (DNA) in exposed cells involved in normal or malignant cells. Fibroblast cell lines repair this DNA damage through histone H2AX phosphorylation in vitro [12]. Although the mechanism of RIS occurrence has not yet been clarified, DNA damage repair might induce some gene variants associated with second malignant neoplasms [13].

We searched the keywords “breast”, “fibrosarcoma, and “radiation” in PubMed in April 2022. We also checked the references cited in the original articles and excluded articles that had no history of radiation therapy, breast cancer and protrubed fibrosarcoma on skin. Thirty cases of radiation-associated fibrosarcoma after breast cancer therapy without protruded cutaneous fibrosarcoma were identified, including our case [14–35] (Table 2). All cases were detected based on the patient’s self or physical findings. They were 52.7 ± 10.3 years old, and the duration from primary breast cancer was 9.4 ± 4.9 years (1.3–17 years, median 8.0 years). Within the description, 12 patients (52%) died, and 10 (48%) were alive. RIS also occurred on post mastectomy state. Although the number of cases was small, we compared dead and alive cases using a *t*-test (Table 3). Not only age, but also duration from primary breast cancer diagnosis, radiation dose, and following months were not significantly different as opposed to prior reports. In recent cases, there might be high accuracy of diagnostic modality and RIS might be detected smaller, diagnosed earlier and lead to more remissions than past cases.

**Table 2:** Cases of fibrosarcoma after radiation therapy for breast cancer

	References	Reported year	Age	Primary breast cancer	Operation	Radiation dose	Duration (year)	Location of fibrosarcoma	Following	Prognosis
1	[14]	1959	63	T1N1	Radical mastectomy	45 Gy	5	Rib	3 years	Death
2	[15]	1968	58	–	Radical mastectomy	+	15	Chest wall, shoulder	–	–
3	[16]	1970	50	–	Radical mastectomy	220 kvp	6	Chest wall, shoulder	1 year	Death
4	[17]	1970	43	Stage III	Radical mastectomy	40 Gy	14	Breast	4 months	Death
5	[18]	1970	53	–	Radical mastectomy	39 Gy	4	Chest wall	3 years	Death
6	[19]	1970	55	–	Radical mastectomy	25 Gy	10	Shoulder	8 months	Death
7			40	–	Radical mastectomy	28 Gy	10	Chest wall	6 months	Alive
8	[20]	1976	57	–	Radical mastectomy	NR	16	Breast, chest wall	–	–
9	[14]	1976	48	T2N0	Simple mastectomy	45 Gy	5.5	Supra-clavian region	2 years	Death
10	[21]	1977	66	–	Radical mastectomy	40 Gy	7	Chest wall	1.5 years	Death
11	[22]	1977	59	–	Radical mastectomy	20 Gy	12	Shoulder, axillary region	2 years	Death
12	[14]	1978	58	T2N1	Simple mastectomy	45 Gy	4	Axillary region	13 years	Alive
13	[23]	1978	63	–	Radical mastectomy	25 Gy	14	Breast	–	–
14	[24]	1981	39	–	Radical mastectomy	40.05 Gy	11	Sternum	3.5 years	Alive
15	[25]	1984	43	–	NR	50 Gy	5	Chest wall	7 months	Death
16	[26]	1986	61	–	Simple mastectomy	30 Gy	17	Supra-clavian region	1 year	Death
17	[27]	1990	66	–	NR	NR	15.5	Chest wall, axillary region	1.8 years	Alive
18	[28]	1994	46	T1N0	Quadrantectomy	60 Gy	1.3	Breast	–	Alive
19	[29]	1996	–	–	NR	NR	7	Chest wall	–	–
20			–	–	NR	NR	8	Axillary region	–	–
21			–	–	NR	NR	17	Pectoral muscle	–	–
22	[30]	1998	–	–	Quadrantectomy	46 + 12 Gy	2	Axillary region	–	Alive
23	[31]	1998	39	T2N2	Tumorectomy	60 Gy	8	Subclavian region	34 months	Death
24			47	T1N0	Tumorectomy	45 Gy	16	Chest wall	18 months	Death
25			40	T2N0	Tumorectomy	60 Gy	7	Axillary region	5 months	Death
26	[32]	2002	42	T2N+	Mastectomy	50 Gy	17	Subclavian region	19 months	Alive
27	[33]	2004	72	–	NR	50 Gy	4	Chest wall	2.8 years	Alive
28	[34]	2006	44	T1N1	Quadrantectomy	50 Gy	2	Axillary region	–	Alive
29	[35]	2013	68	T2N0	Mastectomy	–	6	Spine	–	–
30	Present	2022	43	T1N1	Partial resection	50 Gy	8	Axillary breast	–	Alive

*NR* not reported

**Table 3:** Comparison with cases of dead and alive cases by *t*-test

	Alive *n* = 10	Death *n* = 12	*p* value
Age (years old)	50.0 ± 12.2	51.4 ± 9.4	0.77
Duration (years)	8.1 ± 5.7	9.2 ± 4.5	0.62
Radiation dose (Gy)	47.9 ± 9.6	41.6 ± 12.3	0.22
Following months	40.3 ± 52.9	18.8 ± 12.0	0.26

## Conclusions

Fibrosarcomas after breast-conserving surgery and radiation therapy are rare. Patients with breast masses after radiation therapy should be suspected for not only local recurrence but also RIS.

## Data Availability

All data generated or analyzed during this study are included in this article.

## Abbreviations

RIS: Radiation-induced sarcoma
CNB: Core needle biopsy
DNA: Deoxyribonucleic acid
